# Mass azithromycin distribution for hyperendemic trachoma following a cluster-randomized trial: A continuation study of randomly reassigned subclusters (TANA II)

**DOI:** 10.1371/journal.pmed.1002633

**Published:** 2018-08-14

**Authors:** Jeremy D. Keenan, Zerihun Tadesse, Sintayehu Gebresillasie, Ayalew Shiferaw, Mulat Zerihun, Paul M. Emerson, Kelly Callahan, Sun Y. Cotter, Nicole E. Stoller, Travis C. Porco, Catherine E. Oldenburg, Thomas M. Lietman

**Affiliations:** 1 Francis I. Proctor Foundation, University of California, San Francisco, San Francisco, California, United States of America; 2 Department of Ophthalmology, University of California, San Francisco, San Francisco, California, United States of America; 3 The Carter Center, Ethiopia, Addis Ababa, Ethiopia; 4 The Carter Center, Atlanta, Georgia, United States of America; 5 Department of Epidemiology and Biostatistics, University of California, San Francisco, San Francisco, California, United States of America; The Hospital for Sick Children, CANADA

## Abstract

**Background:**

The World Health Organization recommends annual mass azithromycin administration in communities with at least 10% prevalence of trachomatous inflammation–follicular (TF) in children, with further treatment depending on reassessment after 3–5 years. However, the effect of stopping mass azithromycin distribution after multiple rounds of treatment is not well understood. Here, we report the results of a cluster-randomized trial where communities that had received 4 years of treatments were then randomized to continuation or discontinuation of treatment.

**Methods and findings:**

In all, 48 communities with 3,938 children aged 0–9 years at baseline in northern Ethiopia had received 4 years of annual or twice yearly mass azithromycin distribution as part of the TANA I trial. We randomized these communities to either continuation or discontinuation of treatment. Individuals in the communities in the continuation arm were offered either annual or twice yearly distribution of a single directly observed dose of oral azithromycin. The primary outcome was community prevalence of ocular chlamydial infection in a random sample of children aged 0–9 years, 36 months after baseline. We also assessed the change from baseline to 36 months in ocular chlamydia prevalence within each arm. We compared 36-month ocular chlamydia prevalence in communities randomized to continuation versus discontinuation in a model adjusting for baseline ocular chlamydia prevalence. A secondary prespecified analysis assessed the rate of change over time in ocular chlamydia prevalence between arms. In the continuation arm, mean antibiotic coverage was greater than 90% at all time points. In the discontinuation arm, the mean prevalence of infection in children aged 0–9 years increased from 8.3% (95% CI 4.2% to 12.4%) at 0 months to 14.7% (95% CI 8.7% to 20.8%, *P =* 0.04) at 36 months. Ocular chlamydia prevalence in communities where mass azithromycin distribution was continued was 7.2% (95% CI 3.3% to 11.0%) at baseline and 6.6% (95% CI 1.1% to 12.0%, *P =* 0.64) at 36 months. The 36-month prevalence of ocular chlamydia was significantly lower in communities continuing treatment compared with those discontinuing treatment (*P =* 0.03). Limitations of the study include uncertain generalizability outside of trachoma hyperendemic regions.

**Conclusions:**

In this study, ocular chlamydia infection rebounded after 4 years of periodic mass azithromycin distribution. Continued distributions did not completely eliminate infection in all communities or meet WHO control goals, although they did prevent resurgence.

**Trial registration:**

This study was prospectively registered at clinicaltrials.gov (clinicaltrials.gov NCT01202331).

## Abstract 2

Catherine E Oldenburg and colleagues investigate effects of continuing or stopping antibiotic prophylaxis for chlamydial eye infection.

## Author summary

**Why was this study done?:**

- Trachoma is the leading infectious cause of blindness in the world.
- Although trachoma is disappearing globally, severe trachoma persists in a few districts in Ethiopia.
- Periodic mass azithromycin distribution reduces infection to very low levels, but complete elimination in all communities has proven difficult.

**What did the researchers do and find?:**

- We assessed whether mass azithromycin distributions could be discontinued after 4 years of treatment in communities with severe trachoma in northern Ethiopia.
- A subset of communities that had participated in a previous cluster-randomized trial were re-randomized to continuation or discontinuation of mass azithromycin distribution.
- Ocular chlamydia infection, which causes trachoma, was found to increase over time after mass azithromycin distribution was stopped. Compared to communities where mass azithromycin distribution was continued, communities where distribution was stopped had significantly higher rates of ocular chlamydia infection after 3 years of study.
- Despite 7 years of treatment in communities continuing mass azithromycin distribution, infection was not eliminated in all communities.

**What do these findings mean?:**

- Trachoma may be the first bacterial disease of humans to be eliminated by a public health program; however, these results indicate that stopping mass azithromycin treatment in some severely affected areas is not realistic.
- Alternative strategies for trachoma elimination may be required for the most severely affected areas.

## Introduction

Mass distribution of azithromycin is a key component of the World Health Organization’s SAFE (surgery for trichiasis, antibiotics, facial cleanliness, and environmental improvement) strategy for trachoma control [1–3]. Antibiotics (azithromycin), facial cleanliness, and environmental improvements (e.g., latrinization and water improvements) are interventions aimed at reducing community transmission of the strains of ocular chlamydia that cause trachoma [4,5]. Although facial cleanliness and environmental improvements are associated with decreased clinical trachoma, we have no evidence that facial hygiene or environmental improvements have any effect on ocular chlamydia [6–8]. On the other hand, a single mass azithromycin distribution has been shown to dramatically reduce the prevalence of ocular chlamydia [9,10]. The WHO currently recommends 3 to 5 rounds of annual mass azithromycin distributions for communities in which the district prevalence of trachomatous inflammation–follicular (TF) among 1- to 9-year-old children exceeds 10%, followed by an impact survey to assess whether treatment should be continued. If the district prevalence of TF is below 10% during the impact survey, then community-level surveys should be done, and any communities with a prevalence of TF < 5% should have mass antibiotic treatments discontinued [11].

The WHO recommendation for when to continue mass azithromycin distribution, while extremely valuable to trachoma programs, is based on very limited empirical evidence. Some studies have found evidence to support the existence of an Allee-like effect, in which chlamydial infections fade away if reduced below some low threshold [9,12–15]. In contrast, other studies have found that infection rapidly rebounds once repeated mass azithromycin distributions are stopped [16,17].

In a previous trial (TANA I), we compared repeated annual versus biannual mass azithromycin distributions to determine whether more frequent azithromycin treatment would lead to better control of ocular chlamydia [18]. The trial found a marked reduction in the prevalence of ocular chlamydia among 0- to 9-year-olds in both the annual and biannual groups over the duration of the 3.5-year study, but no significant difference between the 2 treatment strategies at the final study visit [18]. Following the completion of this study, we initiated a continuation trial (TANA II) in which communities from the annual and biannual arms of the original trial were re-randomized to continuing or stopping mass azithromycin distribution to evaluate the need for continued mass azithromycin distributions in areas that have received multiple rounds of mass azithromycin distribution. Here, we report the effect of continuation versus discontinuation of mass azithromycin treatment following 4 years of repeated annual or biannual mass azithromycin distribution on ocular chlamydial infection in a hyperendemic region of Ethiopia.

## Methods

TANA (Trachoma Amelioration in Northern Amhara, ClinicalTrials.gov NCT00322972) was a community-randomized trial that compared different mass azithromycin strategies for reducing ocular chlamydial infection in a hyperendemic area of Ethiopia. The present report describes 4 arms from its continuation trial, Tripartite International Research for the Elimination of Trachoma (TIRET) (ClinicalTrials.gov NCT01202331), also known as and referred to here as TANA II. The trial received ethical approval from the University of California, San Francisco; Emory University; and the Ethiopian Ministry of Science and Technology. Because illiteracy was common among study participants, we obtained verbal informed consent for all study procedures. Caregivers provided verbal consent for children to participate, and community leaders provided consent for their community to participate in the study.

### TANA I overview

**Fig 1:**
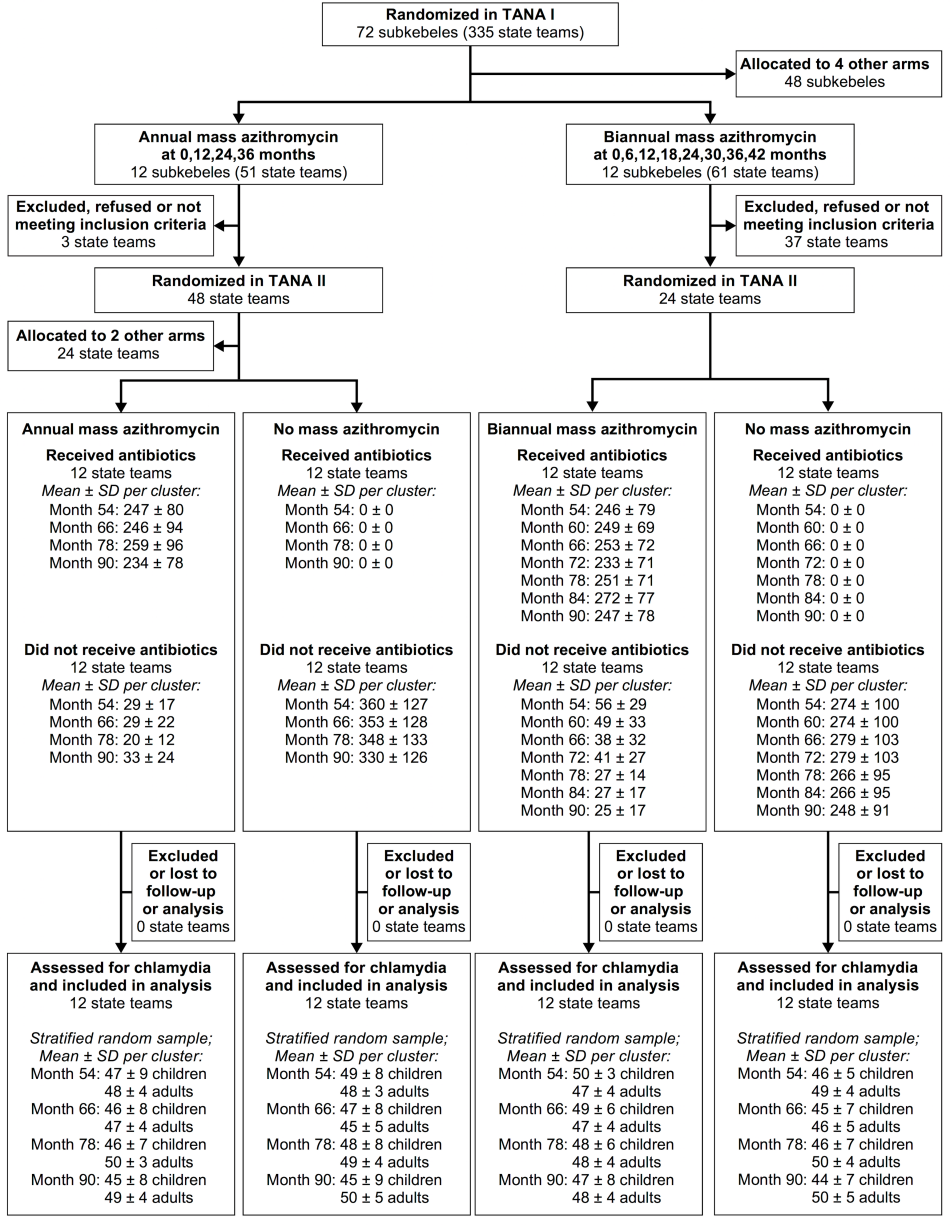
CONSORT diagram. Age range for children is 0–9 years; age range for adults is 10 years and older.

TANA I was a cluster-randomized trial conducted in the Goncha Siso Enese woreda (district) of the Amhara Region of Ethiopia from June 1, 2006, to December 2, 2009 [18]. All communities in the district were eligible for randomization excluding the town of Gindewoin, as it is a semi-urban area with a lower prevalence of trachoma. In the trial, we randomized 72 government-defined subdivisions known as subkebeles to 1 of 6 different treatment strategies, including 12 subkebeles to annual mass azithromycin treatments and 12 subkebeles to biannual mass azithromycin treatments (Fig 1). Subkebeles consisted of 4–6 government-defined units known as state teams (communities). We treated all communities of the subkebele identically, and performed monitoring for ocular chlamydia in a single sentinel state team from the subkebele. We collected conjunctival swabs every 6 months from a random sample of community members, and processed swabs for chlamydia testing using AMPLICOR CT (Roche Molecular Diagnostics, Pleasanton, CA).

### TANA II overview

We conducted the TANA II continuation trial from November 1, 2010, until November 8, 2013. The TANA II trial population consisted of 48 state teams that had received annual mass azithromycin distributions and 24 state teams that had received biannual mass azithromycin distributions during TANA I. State teams were randomly selected stratified by subkebele, with 4 state teams from each of the 12 annually treated subkebeles and 2 state teams from each of the 12 biannually treated subkebeles. We randomized the 4 state teams in each annually treated subkebele to 1 of the following treatment strategies: (1) discontinuation of mass treatments, (2) continued annual mass azithromycin treatments, (3) azithromycin treatments targeted to preschool children, or (4) azithromycin treatments targeted to households containing children with clinically active trachoma. We randomized the 2 state teams from each biannually treated subkebele to either discontinuation of mass treatments or to continued biannual mass azithromycin distributions. As depicted in Fig 1, the present report includes 48 state teams randomized into 4 treatment groups: continuation of annual mass azithromycin (*N =* 12), cessation of annual mass azithromycin (*N =* 12), continuation of biannual mass azithromycin (*N =* 12), and cessation of biannual mass azithromycin (*N =* 12). The 2-stage randomization means that these 4 groups were randomly drawn from the same underlying population and can thus be validly compared with each other. The trial protocol and outcomes were prespecified, and there were no changes to the methods after commencement.

### Randomization and masking

TCP performed the randomization in the statistical package R (R Foundation for Statistical Computing, Vienna, Austria). State teams from the annual and biannual arms of TANA I were randomized to discontinue or continue their treatment strategy. All state teams were enrolled prior to randomization. Study participants were not masked to their state team’s treatment allocation. Field staff were not informed of, and laboratory personnel were masked to, the state team’s treatment allocation.

### Intervention

In both TANA I and TANA II, we performed an annual door-to-door enumerative census. In state teams assigned to treatment, azithromycin was offered to all community members aged 6 months and up (approximately 20 mg/kg using height-based dosing for children, up to a maximum of 1 g) [19]. Children less than 6 months, pregnant women, and those allergic to macrolide antibiotics were offered 2 tubes of topical tetracycline instead, to be used twice daily for 6 weeks. Antibiotic coverage was defined as the proportion of individuals from the most recent census who received either oral azithromycin or topical tetracycline. An adverse event notification system was put in place for individuals to notify the village informant and the Ethiopian study coordinator.

### Monitoring

The primary outcome was prevalence of ocular chlamydia infection as detected by PCR. Active trachoma was a prespecified secondary outcome and is included in this report as it is an important component of monitoring trachoma control programs [20]. We performed annual assessments in the same populations as in TANA I: a random sample of 50 children aged 0–9 years and a random sample of 50 individuals aged 10 years and older (“adults”) from each state team based on the most recent census. To assess active trachoma, at each monitoring visit, we examined the everted upper right tarsal conjunctiva for signs of trachoma according to the WHO simplified trachoma grading system, specifically assessing the presence of TF and trachomatous inflammation–intense. To assess ocular chlamydia, we then collected a conjunctival specimen by passing a Dacron swab 3 times against the everted tarsal conjunctiva, rotating the swab 120° between each pass. We stored swabs on ice for <8 hours while in the field, then at −20°C until transport to the lab, at 4°C for <24 hours during transport, and finally at −80°C until processing. We performed chlamydial testing with the Abbott m2000 platform on pools of 5 randomly selected swabs, with pools stratified by community and age group. We estimated the prevalence of infection most likely to have resulted in the pooled result, as previously described [21].

### Sample size determination

The sample size was based on the primary outcome for the discontinuation and continuation arms, with assumptions based on the results of previous studies (the Trachoma Elimination Follow-up study and TANA I) [18,22]. The standard deviation after 1 year of treatment in TANA I was 0.043, and we conservatively assumed a standard deviation of 0.05 for sample size planning. For assessment of rebound of ocular chlamydia following discontinuation of treatment, a sample size of 12 state teams per arm was determined to provide approximately 80% power to detect a difference of 5% assuming a paired *t* test. For comparison of continued annual versus continued biannual treatment, a sample size of 12 state teams per arm was determined to provide approximately 80% power to detect a 6% difference in ocular chlamydia prevalence.

### Statistical considerations

All analyses were conducted at the level of the unit of randomization, the state team, to account for the clustered design of the trial. We estimated the prevalence of ocular chlamydia and active trachoma in each state team at the community level as described above. The prespecified primary outcome was the prevalence of ocular chlamydia in children aged 0–9 years at 36 months after the baseline visit of TANA II. All analyses were conducted in Stata 14.2 (StataCorp, College Station, TX).

#### Longitudinal changes in prevalence

When analyzing the change in prevalence longitudinally, we analyzed prevalence itself (non-transformed). The prespecified primary analysis was the difference in prevalence from TANA II baseline to 36 months in the discontinuation arm, with communities that had had annual and biannual azithromycin distribution in TANA I aggregated together. In communities randomized to stopping mass azithromycin distribution, we first tested whether the prevalence of ocular chlamydia at 36 months was different from that at the baseline TANA II visit using a paired *t* test. As a prespecified secondary analysis, we modeled chlamydia prevalence over all 4 study visits using a mixed effects linear regression model in which the study community and its study visit were designated as random effects (allowing for a random slope and random intercept for each community’s infection results over time), with treatment arm as a fixed effect. As a secondary analysis, we repeated the analysis with a Wilcoxon signed-rank test. A similar analytic strategy was used to assess the prevalence of ocular chlamydia over time in the continuation arm.

#### Comparison of continuation versus discontinuation

In analyses comparing the 2 arms, we square-root-transformed the prevalence to improve the model fit. Because communities were re-randomized, we included TANA II baseline prevalence as a covariate in models of continuation versus discontinuation of treatment. The prespecified analysis compared the prevalence of infection at 36 months (i.e., 90 months after randomization in TANA I) between the discontinuation and continuation arms in a multiple linear regression with a term for TANA II baseline ocular chlamydia prevalence. As a prespecified sensitivity analysis, we included data from all 4 TANA II monitoring visits in a mixed effects linear regression in which the study community and its study visit were designated as random effects, allowing for a random slope and random intercept for each community’s infection results over time, with treatment arm as a fixed effect. A Monte Carlo permutation test stratified by TANA I study arm with 10,000 replications was used to calculate the *P*-value. As a sensitivity analysis, we compared ocular chlamydia in communities in the continuation and discontinuation arms separately by antibiotic distribution schedule (e.g., annual versus biannual), with an interaction term between TANA I study arm and continuation versus discontinuation. A similar analytic approach was used for TF outcomes.

#### Comparison of annual versus biannual treatment

To compare the results of communities continuing annual versus continuing biannual mass azithromycin distribution, we used a multiple linear regression model with terms for the TANA I baseline value and distribution schedule. Because communities were not re-randomized to annual versus biannual treatment at the start of TANA II, we included the TANA I baseline value: inclusion of the TANA II baseline value would have been a post-randomization correction.

## Results

During the first TANA trial, 12 subkebeles received 4 annual mass azithromycin distributions and 12 subkebeles received 8 biannual mass azithromycin distributions over 3 years, with antibiotic coverage generally exceeding 80% in each community [18]. The results of the TANA I trial have been reported previously [18]. Briefly, the prevalence of ocular chlamydial infection in 0- to 9-year-olds as assessed by AMPLICOR decreased in both of the treatment groups after 3 years of repeated mass azithromycin distributions, from 41.9% to 1.9% in the annual group and from 38.3% to 3.2% in the biannual group. The baseline visit for TANA II took place 54 months after the baseline TANA I visit, which was 18 months after the final mass azithromycin distribution in the annually treated group, and 12 months after the final mass azithromycin distribution in the biannually treated group. Community characteristics for the baseline TANA II visit are shown in Table 1. Antibiotic coverage during TANA II for the 24 communities randomized to continuation of annual or biannual mass azithromycin distribution generally exceeded 80% at each distribution (Table 2). No adverse events were reported in the study.

**Table 1:** Baseline characteristics by study arm.

Characteristic	Discontinuation arm		Continuation arm	
	Annual treatment	Biannual treatment	Annual treatment	Biannual treatment
**Number of state teams**	12	12	12	12
**Population**^1^				
0–9 years	360 (294–430)	274 (222–330)	300 (246–354)	330 (276–391)
≥10 years	90 (73–107)	75 (59–92)	81 (63–98)	93 (77–109)
**Proportion female**^2^				
0–9 years	51.2% (48.2% to 53.9%)	49.5% (45.1% to 53.7%)	48.5% (45.7% to 51.7%)	49.3% (46.7% to 52.1%)
≥10 years	50.2% (48.6% to 51.8%)	53.2% (51.2% to 55.6%)	51.3% (49.5% to 53.0%)	50.8% (49.0% to 52.6%)
**Proportion with TF/TI**^2^				
0–9 years	31.4% (22.3% to 39.8%)	34.5% (23.5% to 46.2%)	42.0% (34.7% to 49.0%)	35.9% (22.7% to 47.8%)
≥10 years	4.6% (1.9% to 9.2%)	4.2% (2.2% to 6.5%)	3.9% (2.2% to 6.1%)	2.9% (1.5% to 5.3%)
**Elevation (m)**^2^	2,585.5 (2,456.3 to 2,679.0)	2,606.4 (2,466.2 to 2,721.2)	2,510.3 (2,354.3 to 2,638.3)	2,531.2 (2,374.2 to 2,682.7)

TF, trachomatous inflammation–follicular; TI, trachomatous inflammation–intense.

^1^Range given in parentheses.

^2^95% confidence interval given in parentheses.

**Table 2:** Antibiotic coverage by study arm.

Time point	Discontinuation arm		Continuation arm	
	Annual treatment	Biannual treatment	Annual treatment^1^	Biannual treatment^1^
0 months	—	—	93.7% (90.6% to 96.3%)	90.5% (87.9% to 93.4%)
6 months	—	—	—	91.0% (88.3% to 93.4%)
12 months	—	—	92.9% (88.7% to 96.7%)	91.4% (88.5% to 94.3%)
18 months	—	—	—	92.8% (90.7% to 95.0%)
24 months	—	—	95.8% (94.3% to 97.2%)	94.3% (91.7% to 96.7%)
30 months	—	—	—	95.0% (93.5% to 96.5%)
36 months	—	—	91.9% (87.3% to 94.3%)	91.7% (87.8% to 94.7%)

^1^95% confidence interval given in parentheses.

Tables 3 and 4 show the prevalence of ocular chlamydia among children aged 0–9 years in each state team over time in the discontinuation and continuation arms, respectively. In the 24 state teams in which mass azithromycin distribution was discontinued, the mean prevalence of ocular chlamydia infection increased from 8.3% (95% CI 4.2% to 12.4%) at TANA II baseline to 14.7% (95% CI 8.7% to 20.8%) at the 36-month visit (*P =* 0.04, paired *t* test). This result was consistent with a repeated measures model that included all time points (*P =* 0.01) and with a Wilcoxon signed-rank test (*P =* 0.054). In contrast, the prevalence of ocular chlamydia did not change markedly in the 24 state teams where mass azithromycin distribution was continued, decreasing from 7.2% (95% CI 3.3% to 11.0%) at TANA II baseline to 6.6% (95% CI 1.1% to 12.0%) at 36 months (*P =* 0.85, paired *t* test), although a repeated measures model including all time points indicated a decrease in prevalence over time (*P =* 0.047).

**Table 3:** Longitudinal prevalence of ocular chlamydia among a random sample of 0- to 9-year-old children after discontinuation of mass azithromycin distribution.

State team	Prevalence of ocular chlamydia, 0- to 9-year-old children			
	0 months	12 months	24 months	36 months
**Annual treatment**				
1	2.0% (1/50)	8.9% (5/56)	3.8% (2/53)	13.9% (5/36)
2	5.7% (3/53)	6.0% (3/50)	15.1% (8/53)	16.0% (8/50)
3	12.7% (7/55)	6.1% (3/49)	18.5%)(10/54)	3.7% (2/54)
4	22.6% (12/53)	21.8% (12/55)	12.5% (6/48)	17.5% (7/40)
5	0.0% (0/46)	8.9% (4/45)	13.0% (7/54)	24.5% (12/49)
6	0.0% (0/51)	0.0% (0/43)	0.0% (0/44)	16.7% (8/48)
7	5.8% (3/52)	0.0% (0/37)	4.3% (2/46)	2.0% (1/50)
8	7.8%(4/51)	2.1% (1/47)	6.1% (3/49)	3.9% (2/51)
9	0.0% (0/26)	0.0% (0/28)	0.0% (0/26)	0.0% (0/21)
10	0.0% (0/52)	0.0% (0/56)	10.9% (6/55)	17.8% (8/45)
11	36.0% (18/50)	34.0% (16/47)	41.7% (20/48)	46.8% (22/47)
12	26.0% (13/50)	11.8% (6/51)	16.3% (8/49)	4.2% (2/48)
Mean (SD)	9.9% (12.1)	8.3% (10.4)	11.9% (11.3)	13.9% (13.0)
**Biannual treatment**				
13	20.0% (8/40)	16.2% (6/37)	34.2% (13/38)	20.0% (8/40)
14	2.3% (1/44)	0.0% (0/43)	0.0% (0/36)	0.0% (0/37)
15	2.0% (1/50)	18.2% (10/55)	26.5% (13/49)	33.3% (17/51)
16	7.8% (4/51)	5.9% (3/51)	20.4% (10/49)	4.0% (2/50)
17	17.4% (8/46)	17.1% (7/41)	27.1% (13/48)	23.3% (10/43)
18	11.8% (6/51)	11.8% (6/51)	27.9% (12/43)	28.9% (11/38)
19	0.0% (0/49)	1.9% (1/52)	2.0% (1/50)	2.0% (1/50)
20	8.8% (3/34)	2.9% (1/35)	0.0% (0/32)	0.0% (0/27)
21	0.0% (0/48)	0.0% (0/52)	0.0% (0/55)	0.0% (0/49)
22	2.0% (1/49)	0.0% (0/44)	2.0% (1/51)	37.3% (19/51)
23	8.0% (4/50)	22.9% (11/48)	30.8% (16/52)	37.5% (18/48)
24	0.0% (0/40)	0.0% (0/34)	0.0% (0/45)	0.0% (0/41)
Mean (SD)	6.7% (6.9)	8.1% (8.6)	14.2% (14.5)	15.5% (16.0)

**Table 4:** Longitudinal prevalence of ocular chlamydia among a random sample of 0- to 9-year-old children with continuation of mass azithromycin distribution.

State team	Prevalence of ocular chlamydia, 0- to 9-year-old children			
	0 months	12 months	24 months	36 months
**Annual treatment**				
25	0.0% (0/32)	5.6% (2/36)	5.9% (2/34)	5.3% (2/38)
26	10.0% (3/30)	0.0% (0/26)	0.0% (0/33)	0.0% (0/29)
27	11.1% (6/54)	2.1% (1/47)	4.8% (2/42)	2.4% (1/41)
28	11.1% (0/51)	16.7% (8/48)	0.0% (0/46)	5.7% (3/53)
29	0.0% (0/50)	0.0% (0/52)	0.0% (0/49)	20.4% (10/49)
30	16.7% (8/48)	0.0% (0/49)	1.9% (1/52)	3.6% (2/55)
31	8.0% (4/50)	0.0% (0/47)	0.0% (0/48)	1.9% (1/54)
32	3.7% (2/54)	2.0% (1/49)	0.0% (0/49)	2.5% (1/40)
33	0.0% (0/33)	0.0% (0/38)	2.4% (1/42)	0.0% (0/38)
34	1.9% (1/52)	0.0% (0/52)	0.0% (0/53)	5.8% (3/52)
35	11.8% (6/51)	6.0% (3/50)	7.7% (4/52)	59.5% (25/42)
36	30.8% (16/52)	11.5% (6/52)	3.9% (2/51)	11.3% (6/53)
Mean (SD)	8.8% (8.9)	3.7% (5.4)	2.2% (2.7)	9.9% (16.6)
**Biannual treatment**				
37	23.4% (11/47)	5.0% (2/40)	7.5% (3/40)	0.0% (0/38)
38	0.0% (0/45)	4.8% (2/42)	0.0% (0/39)	0.0% (0/40)
39	3.6% (2/55)	1.9% (1/53)	0.0% (0/57)	0.0% (0/52)
40	0.0% (0/52)	0.0% (0/52)	0.0% (0/50)	0.0% (0/38)
41	27.7% (13/47)	2.1% (1/48)	20.0% (10/50)	1.9% (1/54)
42	4.3% (2/47)	12.7% (7/55)	3.8% (2/52)	23.4% (11/47)
43	0.0% (0/51)	0.0% (0/53)	0.0% (0/55)	0.0% (0/52)
44	0.0% (0/48)	0.0% (0/38)	0.0% (0/37)	6.3% (2/32)
45	0.0% (0/53)	0.0% (0/52)	0.0% (0/49)	0.0% (0/56)
46	4.0% (2/50)	0.0% (0/49)	0.0% (0/52)	5.8% (3/52)
47	4.0% (2/50)	4.0% (2/50)	8.5% (4/47)	1.9% (1/52)
48	0.0% (0/50)	0.0% (0/51)	0.0% (0/51)	0.0% (0/53)
Mean (SD)	5.6% (9.5)	2.5% (3.8)	3.3% (6.1%)	3.3% (6.7)

At 36 months, there was no significant difference between state teams that had continued to receive annual (9.9%, 95% CI 0% to 20.4%) versus biannual (3.3%, 95% CI 0% to 7.5%) mass azithromycin distributions (*P =* 0.09, linear regression adjusted for TANA I baseline infection prevalence).

**Fig 2:**
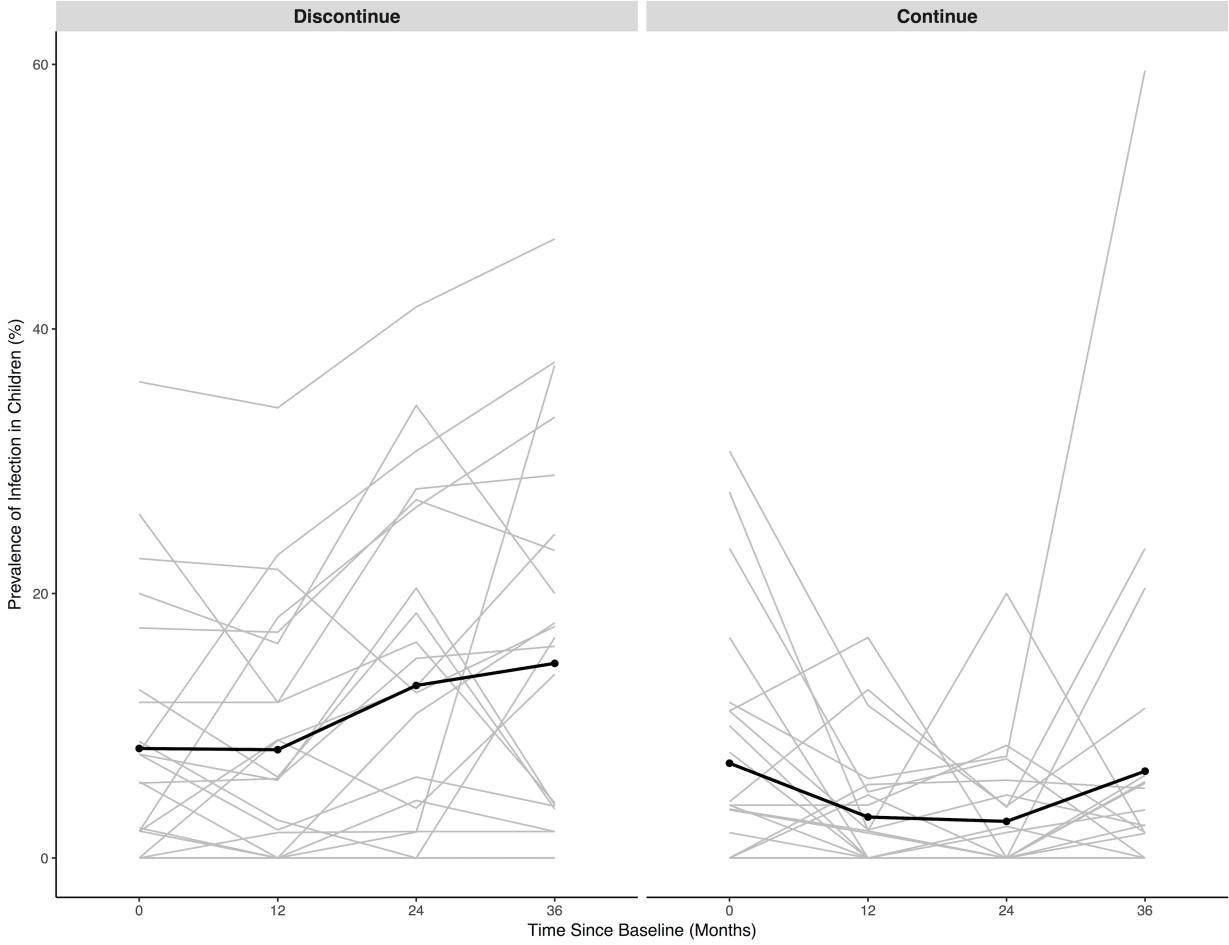
Longitudinal prevalence of ocular chlamydia in children aged 0–9 years. Black line is the mean in all communities. Grey lines are the prevalence in each community.

The 24 state teams randomized to discontinuation of mass azithromycin distributions had a significantly higher prevalence of ocular chlamydia at 36 months (14.7%, 95% CI 8.7% to 20.8%) compared to the 24 state teams that continued treatment (6.6%, 95% CI 1.1% to 12.0%; *P =* 0.03, linear regression adjusted for prevalence of infection at TANA II baseline). In a prespecified sensitivity analysis including data from all time points in a repeated measures model with a time by treatment arm interaction term, state teams randomized to discontinuation had significantly higher ocular chlamydia prevalence over time compared to those that continued mass azithromycin distributions (*P-*interaction *=* 0.02; Fig 2). There was no evidence of effect modification by treatment frequency (annual or biannual) of the effect of continuation versus discontinuation (*P*-interaction = 0.43). However, the reduction in prevalence in the continuation compared to the discontinuation arm was greater in biannually treated communities compared to annually treated communities (*P =* 0.04 biannual, *P =* 0.30 annual).

**Fig 3:**
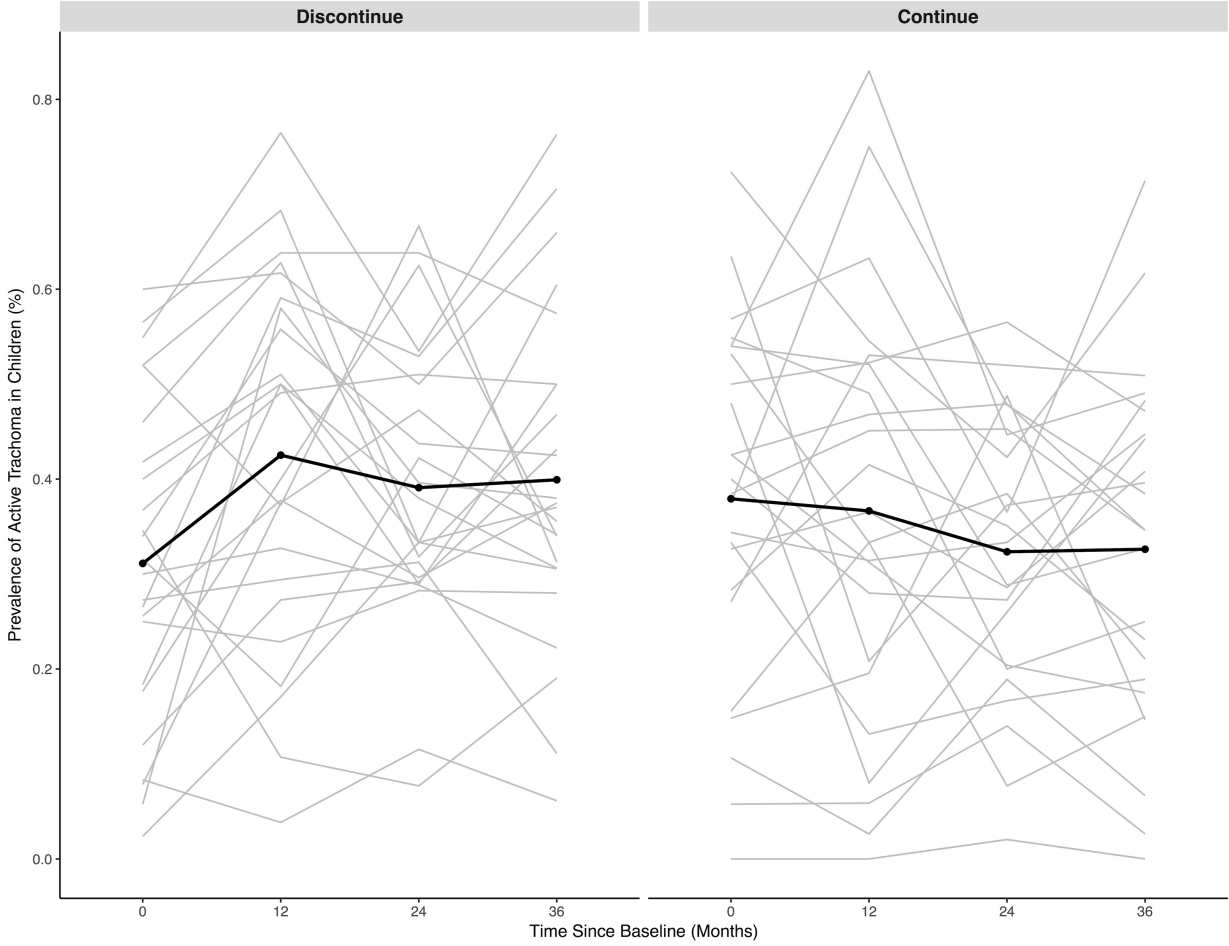
Longitudinal prevalence of trachomatous inflammation–follicular in children aged 0–9 years. Black line is the mean in all communities. Grey lines are the prevalence in each community.

Similar analyses were performed for TF in order to assess changes in clinically active trachoma (S1 and S2 Tables). In state teams in which mass azithromycin distribution was discontinued, the mean prevalence of TF at TANA II baseline was 31.1% (95% CI 23.9% to 38.3%), which increased to 39.9% (95% CI 32.5% to 47.4%) at 36 months (*P =* 0.02, paired *t* test). In communities in which mass azithromycin distributions were continued, the mean TF prevalence at TANA II baseline was 37.9% (95% CI 30.0% to 45.8%), which decreased to 32.6% (95% CI 24.8% to 40.4%) at 36 months (*P =* 0.05, paired *t* test). Communities in which mass azithromycin distributions were discontinued had significantly higher TF prevalence at 36 months compared to communities in which mass azithromycin distributions were continued (mean difference 11.7%, 95% CI 3.5% to 20.0%; *P =* 0.004, linear regression model adjusted for TF prevalence at TANA II baseline). In a repeated measures sensitivity analysis, state teams randomized to discontinuation of mass azithromycin distribution had significantly higher TF prevalence over time (time by treatment *P-*interaction *=* 0.006, mixed effects model; Fig 3). There was no significant difference in change in TF prevalence in communities originally randomized to continuation versus discontinuation in annually and biannually treated communities (*P*-interaction = 0.07), although, similar to ocular chlamydia prevalence, there was a stronger effect of continuation in biannually compared to annually treated communities (*P =* 0.001 biannual, *P =* 0.41 annual).

## Discussion

Although continuation of mass azithromycin distribution led to reduced prevalence of ocular chlamydia compared to communities in which distribution was discontinued, this study demonstrates that even 7 rounds of mass azithromycin were not sufficient for elimination in an area with hyperendemic trachoma. We initially hypothesized that several rounds of mass azithromycin distributions might completely eliminate ocular chlamydia [18]. Previous work with topical tetracycline ointment indicated that antibiotics alone would not be sufficient to achieve trachoma elimination [23]. However, limitations of topical tetracycline, including long treatment duration and poor adherence, may reduce its effectiveness [24]. Single-dose azithromycin can overcome many of these limitations, although previous work has indicated ongoing transmission of ocular chlamydia even after 3 years of implementation of the SAFE strategy in a hyperendemic region of Ethiopia [25]. In the present study, in communities that had previously received 4 years of repeated mass azithromycin distributions, the prevalence of both clinically active trachoma and ocular chlamydia infection stabilized if mass treatments were continued, and increased if mass treatments were stopped. Continuing antibiotic distributions was superior to discontinuing treatments, arguing for continued rounds of antibiotics if the goal is maintaining low levels of ocular chlamydia.

Communities in which treatment was discontinued demonstrated a slower rate of return of infection than previous studies in other hyperendemic regions of Ethiopia. A study in the Gurage zone of Ethiopia found that the average prevalence of ocular chlamydia among 1- to 5-year-old children decreased from 64% to 2.6% after 4 biannual treatments, but returned to 25% 2 years later [26]. In contrast, the communities in the present study experienced slightly less than a 2-fold increase in ocular chlamydia reinfection over 3 years. Several factors may explain these results. Communities in the present study were treated with mass azithromycin distributions for an additional year, which may have resulted in more sustained reduction of infection. Clusters of communities in the same district were initially treated identically in the present study, which would have reduced infection in neighboring communities and could have slowed reemergent infection. We cannot rule out the possibility of differences in the underlying strength of transmission between the Gurage zone and the Amhara Region. As has been seen in other longitudinal assessments, the correlation of infection prevalence over time is low at lower levels of infection [16,27], and thus there was noise in trends in individual community prevalence. Finally, the presence of a secular trend could have reduced reinfection independently from the mass azithromycin treatments.

Mathematical models have suggested that biannual mass distributions might be sufficient to completely eliminate infection, even in severely affected areas where annual treatment is not [5,9,10,28,29]. We did not find that to be the case in this setting. In approximately one-half of the communities, we were able to identify infection from a sample of children even after 7 years of mass treatments. Several possibilities could explain the failure to eliminate trachoma. Infection could have been introduced from people not included in the control program, including those living in neighboring districts or those in the study district who did not participate in the program. Alternatively, relatively poor water and sanitation conditions may have promoted transmission [4,30,31]. Another possibility is that in this hyperendemic setting, even biannual treatment is not enough to completely eliminate infection from those most likely to transmit infection (e.g., preschool children) and that more intensive treatments are necessary specifically for these hyper-transmitters in the community.

Longer-term distribution of mass azithromycin may increase selection for macrolide resistance. Although previous studies have not demonstrated an increase in resistance selection in *Chlamydia trachomatis*, evidence is limited [32,33]. If multiple years of mass azithromycin distribution increase the probability of resistance selection in *C*. *trachomatis*, the efficacy of mass azithromycin distribution may wane over time. Mass distribution of azithromycin for trachoma control has previously been shown to lead to selection for macrolide resistance in non-target organisms including *Streptococcus pneumoniae* and *Escherichia coli*, and resistance declines following cessation of antibiotics [34–37]. Monitoring of macrolide resistance selection in communities receiving long-term mass azithromycin distribution may be important for understanding if multiple rounds of treatment have adverse effects on treatment efficacy.

The results of this study must be considered in the context of several limitations. First, in all communities, infection increased from the end of TANA I until the beginning of TANA II, perhaps in part because of an approximately 6-month lag between the end of TANA I and the beginning of TANA II. Second, this study focused on evaluation of continuation versus discontinuation of mass azithromycin, and did not evaluate combined strategies such as the efficacy of azithromycin in the presence of the facial cleanliness and environmental improvement components of the SAFE strategy. In the study communities, facial cleanliness and environmental improvement initiatives were implemented by the Ethiopian government, but we did not monitor their implementation or community adherence to these components. Note that while facial cleanliness and environmental conditions have been associated with decreases in clinically active trachoma, we have no evidence that facial hygiene or environmental programs have any effect on ocular chlamydial infection [38]. Ongoing work is evaluating the efficacy of facial cleanliness and environmental improvement for trachoma control (ClinicalTrials.gov NCT02754583, Principal Investigator J. Keenan). Third, these results are likely not generalizable to settings with hypo- or mesoendemic trachoma, where infection may be disappearing on its own and may not return after several rounds of mass azithromycin distribution [9,39]. Other hyperendemic areas may have different transmission characteristics, and may thus respond differently after repeated mass azithromycin distribution. The results are, however, generalizable to similar regions of Ethiopia, which has the highest burden of trachoma in the world and which will provide the world’s biggest challenge for trachoma elimination.

Four years of repeated mass azithromycin distributions greatly reduced the prevalence of ocular chlamydia in 0- to 9-year-old children, to approximately 3%. Upon cessation of treatments, infection rebounded. Reducing infection to a low level will not in itself prevent infection from rebounding in this setting. Communities in which annual or biannual mass distributions were continued for an additional 3 years experienced stabilization of infection and clinical disease, suggesting that antibiotics alone may not be enough to completely eliminate infection in severely affected areas. However, continuing mass azithromycin distribution after 3–5 years is significantly better for maintaining low levels of ocular chlamydia than is discontinuing treatment.

## Supporting information

S1 CONSORT Checklist CONSORT checklist. (DOC)

S1 Approvals Institutional review board approvals for TANA II. (PDF)

S1 Protocol Manual of operations and procedures for TANA II. (PDF)

S1 Table Longitudinal prevalence of trachomatous inflammation–follicular among a random sample of 0- to 9-year-old children after discontinuation of mass azithromycin distribution. (DOCX)

S2 Table Longitudinal prevalence of trachomatous inflammation–follicular among a random sample of 0- to 9-year-old children with continuation of mass azithromycin distribution. (DOCX)

## Abbreviation

TF: trachomatous inflammation–follicular
