# Supplementary material for: Mass azithromycin distribution for hyperendemic trachoma following a cluster-randomized trial: A continuation study of randomly reassigned subclusters (TANA II)
Source: PLoS Med. 2018 Aug 14;15(8):e1002633. doi: 10.1371/journal.pmed.1002633 (PMC6091918; doi:10.1371/journal.pmed.1002633)
Supplement: S1 Approvals — (PDF) [file pmed.1002633.s002.pdf]

በኢትዮጵያ ፌዴራላዊ ዴሞክራሲያዊ ሪፐብሊክ
የሳይንስና ቴክኖሎጂ ሚኒስቴር
The Federal Democratic Republic of Ethiopia
Ministry of Science and Technology

ቁጥር 3,10/625/06
Ref. No.
ቀን 26/06/06
Date

To: The Carter Center

Addis Ababa

Re: Tripartite international research for elimination of trachoma (TIRET)

Dear sir/Mr./s/Dr.

We are writing this letter in reference to your amendement request letter dated 30 December, 2013

After having in depth review of your request, National Research Ethics Review committee has accepted your amendment request for one year (**from March 5 / 2014- March 4/ 2015**).

This is, therefore, to notify that the ethical approval is amended and your group can proceed in accordance to the latest approved document. Please ensure that you submit an annual renewal application 30 days prior to expire date and submit periodical reports. We are confident that your esteemed organization will monitor the ethical implication of the project as it is stipulated in the latest approved document.

With regards,

Yohannes Sitotaw

Secretary of NRERC

Cc: _ Dr Zerihun Tadesse (PI)

Addis Ababa, Ethiopia

ማነጋገር ቢያስፈልግዎ
You may Contact

ፖ.ሳ.ቁ.
P.O.Box 2490

አዲስ አበባ ኢትዮጵያ
Addis Ababa, Ethiopia
E-mail most@ethionet.et

ስልክ
Tel. 251-011-4-674353
Web site:-http://www.most.gov.et

ፋክስ
Fax +251-011-4-66 02 41

የኢትዮጵያ ምግብ፣ መድኃኒትና ጤና ክብካቤ
አስተዳደርና ቁጥጥር ባለሥልጣን
FOOD, MEDICINE AND HEALTHCARE
ADMINISTRATION AND CONTROL AUTHORITY OF
ETHIOPIA

ቁጥር 02/6-1/32/14
Ref. No.

ቀን 11 DEC 2014
Date:

To Professor, Thomas M. Lietman (IP) the Carter Center, Ethiopia

Subject: Clinical Trial Authorization

It is hereby certified that the food, Medicine and Health Care Administration and Control Authority, being the authority responsible by the law to authorize and monitor clinical trial conducted in the country (proclamation 661/2009), has officially authorized the conduct of clinical trial entitled ***"Tripartet International Research for the elimination of Trachoma".*** Hence the authority permits the commencement of the trial.

The clinical trial authorization is subject to the following conditions:

- Initiation, progress and termination/end of trial reports shall be submitted to the authority.
- Any adverse events especially serious adverse event/s or deaths and progress report should be reported to the authority.
- The authority shall be informed of any decision to discontinue the clinical trial (if it is found necessary and the reason of such action will be disclosed to the applicant)
- The clinical trial should be conducted according to the protocol and if any amendment required, the amendment should be approved by the authority before implementation.
- The authority shall inspect the trial site at any time for compliance of the trial for Good Clinical Trial Practice (GCP) and the protocol.

With best regards,

CC:

- **Product registration and Licensing Directorate**

EFMHACA

ፋክስ/Fax: 251-1-52 13 92 P.O.Box: 5681 Tel: 251-1-52 41 22/52 41 23 E-mail: regulatory@fmhaca.gov.et
መልስ በሚሰጡበት ጊዜ የእኛን ደብዳቤ ቁጥር ይጥቀሱ
IN REPLY REFER TO OUR Ref. No.

UCSF
University of California
San Francisco

Human Research Protection Program
Institutional Review Board (IRB)

Expedited Review Approval

Principal Investigator
Thomas M. Lietman, M.D.

Co-Principal Investigator
Jeremy Keenan, M.D., M.P.H., Travis C. Porco, Ph.D.

Type of Submission: Continuing Review Submission Form
Study Title: Tripartite International Research for the Elimination of Trachoma

IRB #: 10-02169
Reference #: 204292
Committee of Record: San Francisco General Hospital Panel
Study Risk Assignment: Minimal

Approval Date: 10/29/2017 **Expiration Date:** 10/28/2018

Regulatory Determinations Pertaining to this Approval:

This research satisfies the following condition(s) for the involvement of children:
45 CFR 46.404, 21 CFR 50.51: Research not involving greater than minimal risk.

Parental Permission and Assent:
The permission of one parent or guardian is sufficient.

The research meets all of the conditions of 45 CFR 46.204 for the involvement of pregnant women or fetuses.

The research meets conditions of 45 CFR 46.205 for the involvement of neonates.

This research is not subject to HIPAA rules.

A waiver of the requirement to obtain a signed consent form is acceptable for this study because, as detailed in the application, the research presents no more than minimal risk of harm to subjects and involves no procedures for which written consent is normally required outside of the research context.
The waiver applies to all subjects.

This submission was eligible for expedited review as:
Category 8(c): Renewal of inactive research protocols or protocols that are essentially complete: where the remaining research activities are limited to data analysis

Data analysis phase:
This study is in data analysis and involves no greater than minimal risk for the population being studied.

All changes to a study must receive UCSF IRB approval before they are implemented. Follow the modification request instructions. The only exception to the requirement for prior UCSF IRB review and approval is when the changes are necessary to eliminate apparent immediate hazards to the subject (45 CFR 46.103.b.4, 21 CFR 56.108.a). In such cases, report the actions taken by following these instructions.

Expiration Notice: The iRIS system will generate an email notification eight weeks prior to the expiration of this study's approval. However, it is your responsibility to ensure that an application for continuing review approval has been submitted by the required time. In addition, you are required to submit a study closeout report at the completion of the project.

For a list of all currently approved documents, follow these steps: Go to My Studies and open the study – Click on Informed Consent to obtain a list of approved consent documents and Other Study Documents for a list of other approved documents.

San Francisco Veterans Affairs Medical Center (SFVAMC): If the SFVAMC is engaged in this research, you must secure approval of the VA Research & Development Committee in addition to UCSF IRB approval and follow all applicable VA and other federal requirements. The UCSF IRB website has more information.
