# Supplementary material for: Mass azithromycin distribution for hyperendemic trachoma following a cluster-randomized trial: A continuation study of randomly reassigned subclusters (TANA II)
Source: PLoS Med. 2018 Aug 14;15(8):e1002633. doi: 10.1371/journal.pmed.1002633 (PMC6091918; doi:10.1371/journal.pmed.1002633)
Supplement: S1 Table — (DOCX) [file pmed.1002633.s004.docx]

**S1 Table.** Longitudinal prevalence of trachomatous inflammation, follicular (TF) among a random sample of 0-9-year-old children after discontinuation of mass azithromycin distribution

|  | Prevalence of TF, 0-9-year-old children | | | | | | | |
| --- | --- | --- | --- | --- | --- | --- | --- | --- |
| Community | Time 0 | | Time 12 | | Time 24 | | Time 36 | |
| Annual |  |  |  |  |  |  |  |  |
| 1 | 30.0% | (15/50) | 32.7% | (18/55) | 28.8% | (15/52) | 22.2% | (8/36) |
| 2 | 5.8% | (3/52) | 58.0% | (29/50) | 39.6% | (21/53) | 38.0% | (19/50) |
| 3 | 41.8% | (23/55) | 51.0% | (25/49) | 33.3% | (18/54) | 37.0% | (20/54) |
| 4 | 34.0% | (18/53) | 55.8% | (29/52) | 43.8% | (21/48) | 42.5% | (17/40) |
| 5 | 25.6% | (11/43) | 37.8% | (17/45) | 29.6% | (16/54) | 37.5% | (18/48) |
| 6 | 46.0% | (23/50) | 62.8% | (27/43) | 31.8% | (14/44) | 46.8% | (22/47) |
| 7 | 25.0% | (13/52) | 22.9% | (8/35) | 28.3% | (13/46) | 28.0% | (14/50) |
| 8 | 12.0% | (6/50) | 27.3% | (12/44) | 29.2% | (14/48) | 43.1% | (22/51) |
| 9 | 34.6% | (9/26) | 10.7% | (3/28) | 7.7% | (2/26) | 19.0% | (4/21) |
| 10 | 7.8% | (4/51) | 37.5% | (21/56) | 47.3% | (26/55) | 35.6% | (16/45) |
| 11 | 52.0% | (26/50) | 63.8% | (30/47) | 63.8% | (30/47) | 57.4% | (27/47) |
| 12 | 52.0% | (26/50) | 37.3% | (19/51) | 66.7% | (32/48) | 31.3% | (15/48) |
| **Mean (SD)** | **30.5% (16.1)** | | **41.5% (16.9)** | | **37.5% (16.3)** | | **36.5% (10.6)** | |
| Biannual |  |  |  |  |  |  |  |  |
| 13 | 40.0% | (16/40) | 40.0% | (16/40) | 28.9% | (11/38) | 50.0% | (20/40) |
| 14 | 2.4% | (1/42) | 2.4% | (1/42) | 33.3% | (12/36) | 30.6% | (11/36) |
| 15 | 36.7% | (18/49) | 36.7% | (18/49) | 51.0% | (25/49) | 50.0% | (25/50) |
| 16 | 17.6% | (9/51) | 17.6% | (9/51) | 62.5% | (30/48) | 34.0% | (17/50) |
| 17 | 56.5% | (26/46) | 56.5% | (26/46) | 33.3% | (16/48) | 60.5% | (26/43) |
| 18 | 54.9% | (28/51) | 54.9% | (28/51) | 53.5% | (23/43) | 76.3% | (29/38) |
| 19 | 18.4% | (9/49) | 18.4% | (9/49) | 38.0% | (19/50) | 30.6% | (15/49) |
| 20 | 27.3% | (9/33) | 27.3% | (9/33) | 31.3% | (10/32) | 11.1% | (3/27) |
| 21 | 8.3% | (4/48) | 8.3% | (4/48) | 11.5% | (6/52) | 6.1% | (3/49) |
| 22 | 26.5% | (13/49) | 26.5% | (13/49) | 52.9% | (27/51) | 70.6% | (36/51) |
| 23 | 60.0% | (30/50) | 60.0% | (30/50) | 50.0% | (26/52) | 66.0% | (31/47) |
| 24 | 31.6% | (12/38) | 31.6% | (12/38) | 42.2% | (19/45) | 34.1% | (14/41) |
| **Mean (SD)** | **31.7% (18.8)** | | **43.6% (22.4)** | | **40.7% (14.1)** | | **43.3% (22.7)** | |
