# Supplementary material for: Mass azithromycin distribution for hyperendemic trachoma following a cluster-randomized trial: A continuation study of randomly reassigned subclusters (TANA II)
Source: PLoS Med. 2018 Aug 14;15(8):e1002633. doi: 10.1371/journal.pmed.1002633 (PMC6091918; doi:10.1371/journal.pmed.1002633)
Supplement: S2 Table — (DOCX) [file pmed.1002633.s005.docx]

**S2 Table.** Longitudinal prevalence of trachomatous inflammation, follicular (TF) among a random sample of 0-9-year-old children with continuation of mass azithromycin distribution

|  | Prevalence of TF, 0-9-year-old children | | | | | | | |
| --- | --- | --- | --- | --- | --- | --- | --- | --- |
| Community | Time 0 | | Time 12 | | Time 24 | | Time 36 | |
| Annual |  |  |  |  |  |  |  |  |
| 25 | 34.4% | (11/32) | 31.4% | (11/35) | 33.3% | (10/30) | 44.7% | (17/38) |
| 26 | 40.0% | (12/30) | 28.0% | (7/25) | 27.3% | (9/33) | 48.3% | (14/29) |
| 27 | 14.8% | (8/54) | 19.6% | (9/46) | 48.8% | (20/41) | 14.6% | (6/41) |
| 28 | 50.0% | (27/54) | 52.3% | (23/44) | 56.5% | (26/46) | 47.2% | (25/53) |
| 29 | 32.7% | (16/49) | 36.5% | (19/52) | 28.6% | (14/49) | 40.8% | (20/49) |
| 30 | 27.1% | (13/48) | 53.1% | (26/49) | 52.0% | (26/50) | 50.9% | (28/55) |
| 31 | 54.0% | (27/50) | 83.0% | (39/47) | 44.7% | (21/47) | 49.1% | (26/53) |
| 32 | 42.6% | (23/54) | 31.3% | (15/48) | 20.4% | (10/49) | 17.5% | (7/40) |
| 33 | 33.3% | (11/33) | 13.2% | (5/38) | 16.7% | (7/42) | 18.9% | (7/37) |
| 34 | 38.5% | (20/52) | 45.1% | (23/51) | 45.3% | (24/53) | 34.6% | (18/52) |
| 35 | 56.9% | (29/51) | 63.3% | (31/49) | 36.5% | (19/52) | 71.4% | (30/42) |
| 36 | 63.5% | (33/52) | 20.8% | (10/48) | 37.3% | (19/51) | 39.6% | (21/53) |
| **Mean (SD)** | **40.6% (14.0)** | | **39.8% (20.3)** | | **37.3% (12.6)** | | **39.8% (16.4)** | |
| Biannual |  |  |  |  |  |  |  |  |
| 37 | 53.2% | (25/47) | 33.3% | (13/39) | 38.5% | (15/39) | 21.1% | (8/38) |
| 38 | 15.6% | (7/45) | 33.3% | (14/42) | 7.7% | (3/39) | 15.0% | (6/40) |
| 39 | 28.3% | (15/53) | 41.5% | (22/53) | 35.1% | (20/57) | 23.1% | (12/52) |
| 40 | 5.8% | (3/52) | 5.9% | (3/51) | 14.0% | (7/50) | 2.6% | (1/38) |
| 41 | 42.6% | (20/47) | 46.8% | (22/47) | 47.9% | (23/48) | 34.6% | (18/52) |
| 42 | 72.3% | (34/47) | 54.5% | (30/55) | 42.3% | (22/52) | 61.7% | (29/47) |
| 43 | 54.9% | (28/51) | 49.1% | (26/53) | 20.0% | (11/55) | 25.0% | (13/52) |
| 44 | 10.6% | (5/47) | 2.6% | (1/38) | 18.9% | (7/37) | 6.7% | (2/30) |
| 45 | 0.0% | (0/52) | 0.0% | (0/52) | 2.0% | (1/49) | 0.0% | (0/55) |
| 46 | 54.0% | (27/50) | 52.1% | (25/48) | 28.8% | (15/52) | 32.7% | (17/52) |
| 47 | 37.5% | (18/48) | 75.0% | (36/48) | 47.8% | (22/46) | 38.5% | (20/52) |
| 48 | 48.0% | (24/50) | 8.0% | (4/50) | 26.0% | (13/50) | 44.2% | (23/52) |
| **Mean (SD)** | **35.2% (23.0)** | | **33.5% (24.3)** | | **27.4% (15.3)** | | **25.4% (18.2)** | |
